# T‐DNA orientation, distance between two T‐DNAs, and the transformation target cells significantly impact vector backbone integration and efficiency of generating marker‐free transgenic plants in a co‐transformation system

**DOI:** 10.1111/tpj.70510

**Published:** 2025-10-07

**Authors:** Mariam Al Nuaimi, Mohammed Rafi, Mohamed ElSiddig, Maitha Aldarmaki, Suja George, Khaled M.A. Amiri

**Affiliations:** ^1^ Khalifa Center for Genetic Engineering and Biotechnology Al‐Ain 15551 United Arab Emirates; ^2^ Department of Biology, College of Science United Arab Emirates University Al Ain 15551 United Arab Emirates

**Keywords:** marker‐free transgenic plants, co‐transformation, vector backbone integration, transformation target cells, T‐DNA orientation

## Abstract

The development of marker‐free transgenic plants is essential to address biosafety concerns and facilitate regulatory approval. Co‐transformation strategies involving separate T‐DNAs for the gene of interest and selectable marker gene offer a clean approach but are often hampered by linked integration and vector backbone incorporation. In this study, we designed and evaluated a series of double T‐DNA vectors with varying intervening sequence lengths and orientations to determine their impact on co‐transformation efficiency and integration patterns in different plant species. Our results showed that shorter spacer regions increased the likelihood of linked T‐DNA integration, while an ~3 kb intervening region minimized this risk. Contrary to previous findings, inverse orientation of T‐DNAs with respect to each other in the vector significantly increased the frequency of linked and closely spaced integrations compared to tandem arrangements. Co‐transformation efficiency and integration outcomes varied across species and transformation methods, with Arabidopsis exhibiting higher rates of linked integration possibly due to germline transformation via floral dip, in contrast to somatic cell transformation in tobacco, lettuce, and tomato. Incorporation of a GFP reporter gene within the intervening region enabled easy identification of unlinked integration events in the T0 generation, reducing downstream screening efforts. Marker‐free plants were successfully recovered in the T1 generation, confirming the effectiveness of this approach. These findings emphasize the importance of T‐DNA design, orientation, and target cell type in optimizing co‐transformation strategies for generating marker‐free transgenic plants.

## Significance Statement

This study offers valuable insights into the molecular determinants of T‐DNA integration patterns during Agrobacterium‐mediated co‐transformation with double T‐DNA vectors. By elucidating the impact of T‐DNA orientation, proximity, and target cell type, it contributes to the refinement of strategies for generating marker‐free transgenic plants.

## INTRODUCTION

The introduction of foreign genes carrying traits of agronomic significance is a commonly employed approach to develop new plant varieties with desirable traits (Kamthan et al., 2016; Kumar et al., 2020; Mackelprang & Lemaux, 2020). Genetic transformation methods, including *Agrobacterium tumefaciens*‐mediated transformation and direct gene transfer techniques, frequently rely on selectable marker genes (SMGs) to identify and select transgenic material (Miki & McHugh, 2004; Súnico et al., 2024; Ziemienowicz, 2001). However, once transgenic plants containing the target gene are successfully regenerated and confirmed, the marker genes become redundant. The persistent presence of SMGs in the final plant raises ecological and biosafety concerns, particularly regarding the potential for horizontal gene transfer to wild species, non‐GM crops, or pathogens (Teng, 2008). This possibility, along with concerns about human health impacts, fuels public resistance to genetically modified (GM) crops (Teng, 2008; Turnbull et al., 2021). Unlike genome‐edited crops, where marker genes can be segregated from mutations in subsequent generations, transgenic plants remain subject to stringent regulations, which can delay their approval and acceptance (Gu et al., 2021). Additionally, the presence of unnecessary marker genes may impose a metabolic load on the plant (Li et al., 2009), while the limited availability of commonly used SMGs further restricts gene stacking and re‐transformation efforts (Zhao et al., 2019). Therefore, developing marker‐free transgenic plants is highly desirable to facilitate their regulatory approval and enhance commercial adoption.

Various approaches have been employed to develop marker‐free transgenic plants with differing levels of success. These include co‐transformation, where the gene of interest (GOI) and SMGs are introduced on separate T‐DNAs, recombination‐based systems that facilitate the excision of SMGs from the plant genome after transformation, and transposon‐based strategies, which enable the physical separation of the SMG from the GOI, allowing their segregation in subsequent plant generations (Tuteja et al., 2012; Woo et al., 2015).

Co‐transformation, where the GOI and SMG are introduced on separate T‐DNAs and later segregated, offers a clean approach that avoids residual DNA sequences in sexually propagated transgenic plants (Liu et al., 2020; Miller et al., 2002). However, its success depends on the independent integration of the two T‐DNAs into unlinked genomic loci, which is inherently less efficient than single T‐DNA integration (Radchuk et al., 2005). Co‐transformation can be achieved through three systems: the double T‐DNA vector system (both T‐DNAs in a single plasmid within one Agrobacterium), the one strain/two plasmids system (separate plasmids in the same Agrobacterium), and the mixed‐strain system (separate plasmids in different Agrobacterium cells) (Liu et al., 2020; McCormac et al., 2001). While studies suggest that co‐transformation effectively segregates marker genes, with the double T‐DNA system being more efficient than the mixed‐strain approach, challenges remain (Daley et al., 1998; Komari et al., 1996; Liu et al., 2020; McCormac et al., 2001; Miller et al., 2002). The low probability of both T‐DNAs integrating into the same cell, coupled with the risk of multiple marker gene insertions (Huang et al., 2004), reduces the likelihood of completely segregating the SMG in subsequent generations (Angenon & Chong‐Pérez, 2013; Tuteja et al., 2012). Furthermore, Agrobacterium‐mediated transformation often results in the unintended integration of vector backbone (VB) sequences due to read‐through beyond T‐DNA borders (De Buck et al., 2000), and in double T‐DNA systems, this can lead to linked integration of both T‐DNAs as a single fragment. To improve the efficiency of marker‐free transgenic plant recovery, an ideal co‐transformation system should promote single‐copy insertions of both T‐DNAs, minimize their linked integration, and incorporate reliable detection methods to eliminate undesired events.

In this study, we developed double T‐DNA vectors incorporating an origin of replication known to enhance single‐copy insertions (Ye et al., 2011; Zhang et al., 2019). To optimize their design, we tested three versions with varying intervening sequence lengths to determine the optimal distance that minimizes the risk of linked T‐DNA integration. We further examined how the relative orientation of the T‐DNAs influences co‐transformation efficiency and integration patterns. Additionally, we evaluated the utility of incorporating a reporter gene within the intervening region to facilitate early identification of unlinked integration events in the T0 generation. We also assessed species‐specific differences in co‐transformation efficiency and linked T‐DNA integration, highlighting the impact of transformation target cell type on integration outcomes.

Overall, our study led to the development of an efficient double T‐DNA vector system that allows early identification of unlinked T‐DNA insertions in the T0 generation, significantly reducing the number of plants required for screening in the T1 generation. This streamlined approach improves efficiency by saving time, space, and labor in the identification of marker‐free transgenic plants.

## RESULTS

### The likelihood of linked integration is influenced by the distance between the T‐DNAs in the vector

**Figure 1:**
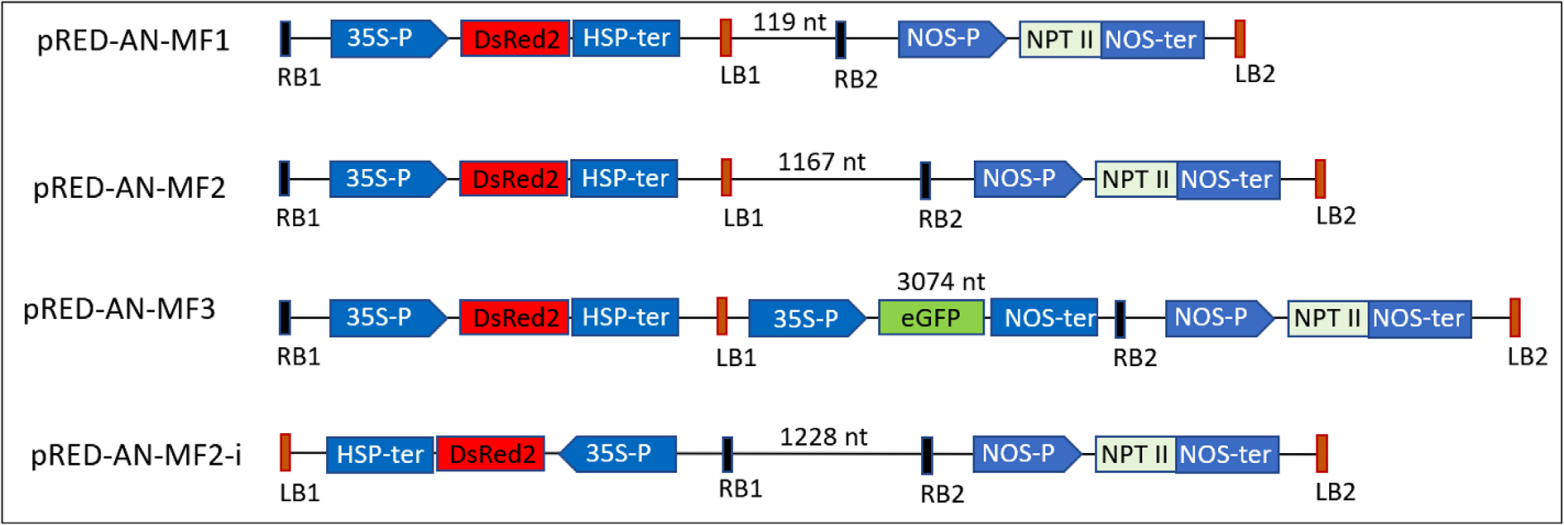
Vector maps of the four double T‐DNA vectors used in this study. T‐DNA1 carries the gene of interest (DsRED2), while T‐DNA2 contains the selectable marker gene (NPT II). In the vector pRED‐AN‐MF3, the spacer region between the two T‐DNAs includes a GFP expression cassette. The length of the spacer region is indicated alongside each construct. RB and LB represent the right and left borders of each T‐DNA, respectively. 35S‐P, CaMV 35S promoter; HSP‐ter, *Arabidopsis thaliana* heat shock protein 18.2 gene terminator; NOS‐P/NOSter, Nopaline synthase promoter and terminator.

**Figure 2:**
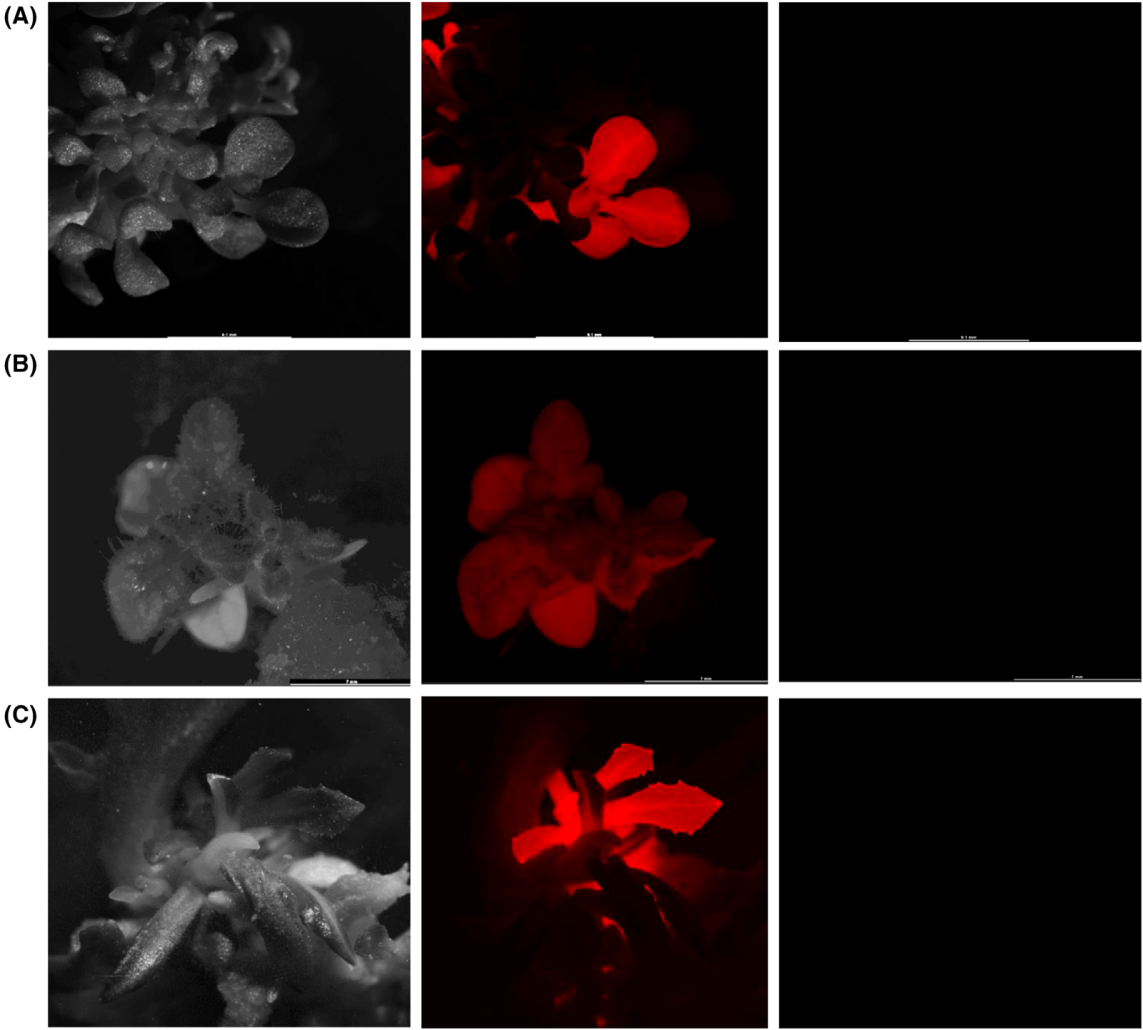
Fluorescence‐based identification of co‐transformed plants in the T_0_ generation. Transgenic shoots regenerated on selection medium following transformation with the vector pRED‐AN‐MF3 were analyzed for RFP fluorescence, enabling the identification of co‐transformation events involving both the gene of interest (GOI, DsRED) and the selectable marker gene (SMG, NPTII). (A) Tobacco, (B) tomato, (C) lettuce.

To assess the impact of intervening region length on the integration of two T‐DNAs as a single unit (linked integration), we designed three double T‐DNA vectors with varying spacer lengths: 116 nt in pRED‐AN‐MF1, 1167 nt in pRED‐AN‐MF2, and 3074 nt in pRED‐AN‐MF3. Here, T‐DNA1 carried GOI (DsRED2) and T‐DNA2 carried SMG (NPT II, conferring kanamycin resistance). Notably, in all three vectors, both T‐DNAs were of similar size (T‐DNA1: 2382 nt; T‐DNA2: 2067 nt) and arranged in a tandem orientation (RB1‐LB1‐RB2‐LB2) (Figure 1). The same Agrobacterium strain (LBA4404) was used for tobacco transformations with these vectors. Since the kanamycin concentration in the selection media was optimized to completely inhibit the regeneration of non‐transgenic shoots, any well‐formed shoots were assumed to have integrated T‐DNA2 carrying the SMG. To assess the co‐transformation efficiency of both T‐DNAs, 100 or more randomly selected shoots were analyzed for RFP fluorescence (Figure 2).

Among the shoots regenerated after transformation with pRED‐AN‐MF1, 57% exhibited red fluorescence, indicating successful co‐transformation. Similar co‐transformation frequencies were observed for pRED‐AN‐MF2 (55%) and pRED‐AN‐MF2‐i (43%), while pRED‐AN‐MF3 showed the lowest efficiency at 19% (Table 1). A chi‐squared test confirmed a highly significant association between vector construct and co‐transformation frequency (*χ* ^2^(3) = 58.87, *P* = 1.03 × 10^−12^). Post hoc pairwise comparisons revealed that co‐transformation frequency with pRED‐AN‐MF3 was significantly lower than with pRED‐AN‐MF1 (*P* = 3.7 × 10^−10^), pRED‐AN‐MF2 (*P* = 2.9 × 10^−9^), and pRED‐AN‐MF2‐i (*P* = 1.1 × 10^−4^), while no significant differences were observed among the other constructs.

**Table 1:** Co‐transformation and linked integration frequencies in T0 tobacco plants transformed with the four double T‐DNA vectors: Subscript next to the vector names indicates the length of the intervening region between the two T‐DNAs

Vector/Agrobacterium strain	T0 plants analyzed	GOI−/SMG+	GOI+/SMG+	Co‐transformation frequency (%)	Linked integration frequency (%)
pRED‐AN‐MF1/LBA4404 _(119 nt)_	100	43	57	57^a^	35^a^
pRED‐AN‐MF2/LBA4404 _(1167 nt)_	100	45	55	55^a^	17^b^
pRED‐AN‐MF3/LBA4404 _(3074 nt)_	200	162	38	19^b^	0^c^
pRED‐AN‐MF2‐i/LBA4404 _(1228 nt)_	100	57	43	43^a^	47^a^

Co‐transformation frequency was calculated as the percentage of regenerated T_0_ shoots exhibiting both GOI and SMG expression (GOI+/SMG+), while linked integration frequency was defined as the percentage of co‐transformants that were PCR‐positive for the intervening spacer region between the two T‐DNAs. Superscript letters indicate statistically significant differences among constructs based on pairwise proportion tests with Bonferroni correction (*P* < 0.05). Frequencies sharing the same letter are not significantly different from each other.

T0 shoots showing co‐transformation were selected and grown further. Genomic DNA was extracted, and samples were first screened for Agrobacterium contamination and plasmid carryover, and any positive samples were excluded. Co‐transformed plants were then genotyped for T‐DNA1 and T‐DNA2, confirming the presence of both GOI and SMG. To assess linked integration, PCR was performed using a forward primer in T‐DNA1 and a reverse primer in T‐DNA2.

PCR analysis of regenerated T0 plants revealed that 35% of pRED‐AN‐MF1 and 17% of pRED‐AN‐MF2 transformants carried the intervening region, confirming linked integration. In contrast, none of the 38 tested pRED‐AN‐MF3 plants showed amplification of the intervening sequence, suggesting a 0% linked integration rate. These findings were consistent with fluorescence screening, as none of the 200 pRED‐AN‐MF3 transformants exhibited GFP expression—indicative of spacer region integration. However, localized patches of GFP expression were observed in some callus tissue (Figure S1). A Pearson's chi‐squared test supported a strong association between vector construct and linked integration frequency (*χ* ^2^(3) = 111.01, *P* < 2.2 × 10^−16^). Pairwise tests confirmed that the linked integration frequency of pRED‐AN‐MF3 was significantly lower than those of pRED‐AN‐MF1 (*P* < 2 × 10^−16^), pRED‐AN‐MF2 (*P* = 5.7 × 10^−8^), and pRED‐AN‐MF2‐i (*P* < 2 × 10^−16^). The highest linked integration was observed for pRED‐AN‐MF2‐i (47%).

While genotyping the spacer region, some transgenic lines exhibited PCR amplicons of unexpected sizes, suggesting unlinked but closely integrated T‐DNAs. Sequencing confirmed that the two T‐DNAs had integrated as separate units in close proximity, sometimes with little to no tobacco genomic sequences between them (Figure S3).

### Orientation of two T‐DNAs influences co‐transformation efficiency and their linked integration

To determine whether the orientation of T‐DNAs in the vector influences their co‐transformation and integration as a single unit, we developed a new vector, pRED‐AN‐MF2‐i, in which the two T‐DNAs are arranged in an inverse orientation (LB1‐RB1‐RB2‐LB2). This was compared to pRED‐AN‐MF2, where the T‐DNAs are in a tandem orientation (RB1‐LB1‐RB2‐LB2), while keeping the length of the intervening region nearly identical (Figure 1).

**Figure 3:**
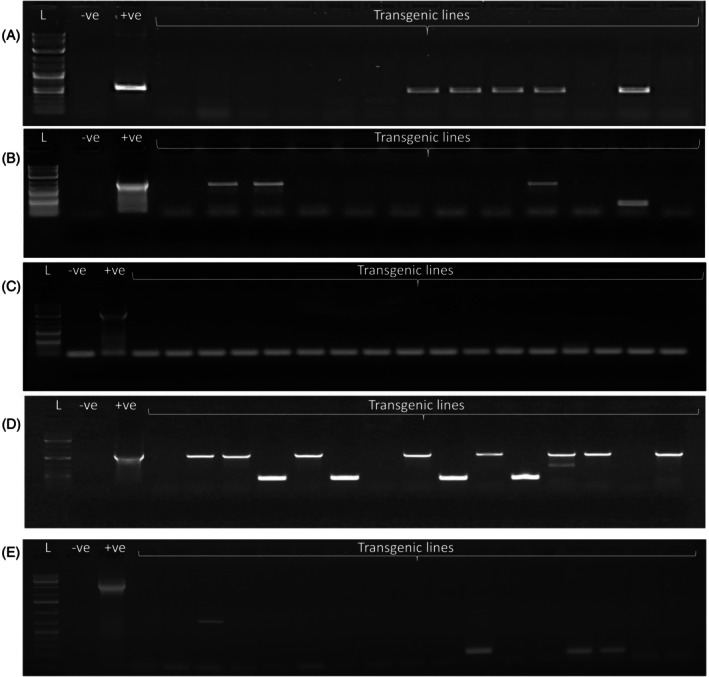
PCR genotyping of T0 tobacco plants to detect the integration of the two T‐DNAs as a single unit (linked integration) following transformation with various vectors using *Agrobacterium tumefaciens* strain LBA4404. (A) pRED‐AN‐MF 1, (B) pRED‐AN‐MF 2, (C) pRED‐AN‐MF 3, (D) pRED‐AN‐MF 2‐i, (E) pRED‐AN‐MF 3 using Agrobacterium strain GV3101. L, DNA size ladder; −ve, negative control (non‐transformed plant DNA); +ve, positive control (respective plasmid DNA). Representative transgenic lines are shown.

Tobacco explants were transformed with Agrobacterium strain LBA4404, and transgenic pRED‐AN‐MF2‐i tobacco shoots regenerated on selection media were analyzed for RFP fluorescence. 43% of the shoots displayed RFP, indicating co‐transformation of both T‐DNAs. This co‐transformation rate was lower than that observed for pRED‐AN‐MF2 (55%), in which the T‐DNAs are arranged in a tandem orientation. PCR analysis of 30 co‐transformed T0 pRED‐AN‐MF2‐i transgenic plants confirmed the presence of both the GOI and SMG in all samples. When these plants were genotyped for the presence of the intervening region, 14 plants produced an amplicon of the expected size, indicating that nearly 50% had undergone linked integration. This is significantly higher than the 17% linked integration observed in pRED‐AN‐MF2 transgenic plants, where the T‐DNAs are arranged in tandem orientation (Figure 3, Table 1). The number of transgenic lines showing amplicons of unexpected sizes for the intervening region was also higher for vector pRED‐AN‐MF2‐i. Random sequencing indicated that these lines have integrated both T‐DNAs individually, but close to each other.

These results indicate that transformation with pRED‐AN‐MF2‐i reduced co‐transformation efficiency while increasing the frequency of linked integration and close individual integration events, emphasizing the significant impact of T‐DNA orientation on co‐transformation and integration patterns.

### Species‐specific differences in co‐transformation efficiency and T‐DNA integration

We evaluated the co‐transformation efficiency of two T‐DNAs across different plant species, including lettuce, tomato, and Arabidopsis. Since pRED‐AN‐MF3 exhibited no instances of linked integration in tobacco, we selected this vector for the study. Agrobacterium strain LBA4404 carrying pRED‐AN‐MF3 was used for transforming lettuce and tomato, while the transformation of Arabidopsis was conducted with Agrobacterium strain GV3101, as this strain has been reported to achieve higher transformation efficiencies in Arabidopsis floral dip transformation (Clough & Bent, 1998; Ghedira et al., 2013).

Transgenic lettuce and tomato plants were regenerated using selection media optimized to suppress the growth of non‐transgenic shoots. Initially, the transformation of lettuce with the vector pRED‐AN‐MF3 failed to produce any transgenic shoots. To overcome this, we used a modified version of the vector in which the original SMG cassette was replaced with that from vector PKSE401. This replacement cassette included the NPT II gene driven by an enhanced CaMV 35S promoter and a polyadenylation signal. Transformation with the modified vector enabled successful shoot regeneration on selection media. Based on RFP fluorescence, the co‐transformation efficiency was 13% in tomato and 9% in lettuce, compared to the 19% observed in tobacco (Figure 2). GFP fluorescence was not detected in any regenerated shoots, suggesting that linked integration events were likely absent in these lines. Although the absence of fluorescence does not completely rule out the possibility of non‐expressed or truncated GFP integrations without further PCR validation, the results nonetheless support a high frequency of successful segregation between the two T‐DNAs following transformation with pRED‐AN‐MF3 in these species.

**Figure 4:**
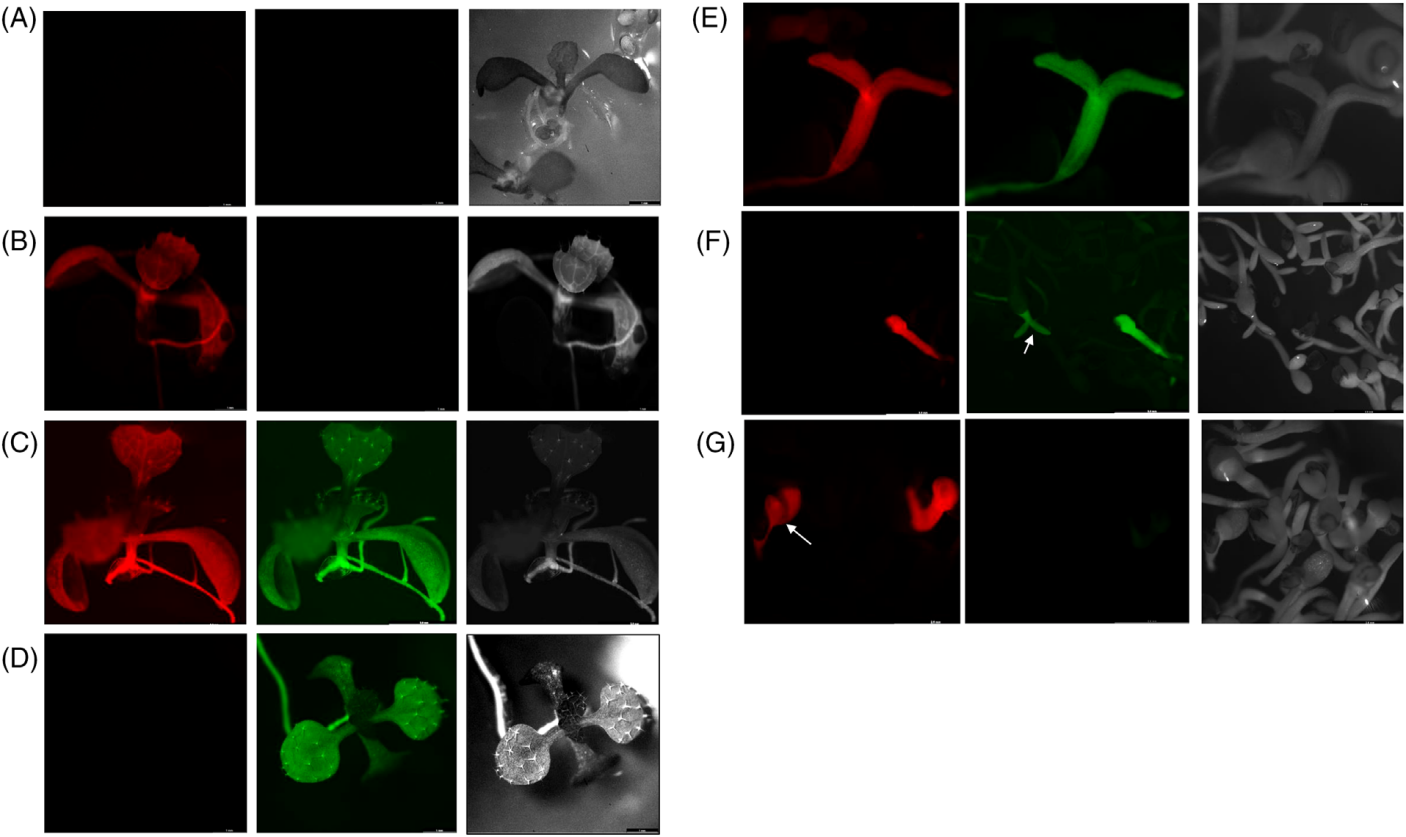
Patterns of flourescence observed in Arabidopsis after transformation with pRED‐ANMF3 using Agrobacterium strain GV3101 indicating different integration events. (A) T‐DNA2 alone (GOI−/GFP−/SMG+), (B) T‐DNA1 and T‐DNA2 integrated separately (GOI+/GFP−/SMG+), (C) T‐DNA1 and T‐DNA2 integrated as a single unit (GOI+/GFP+/SMG+), (D) T‐DNA2 and the intervening region without complete integration of T‐DNA1 (GOI−/GFP+/SMG+), (E) T‐DNA1 and the intervening region without complete integration of T‐DNA2 (GOI+/GFP+/SMG−), (F) integration of the intervening region alone without complete integration of T‐DNA1 or T‐DNA2 (GOI−/GFP+/SMG−), (G) T‐DNA1 alone (GOI+/GFP−/SMG−).

In Arabidopsis, seeds harvested after floral dip transformation were germinated on selection media. Of the 158 seedlings that survived selection, 56 showed no fluorescence, indicating transformation of only T‐DNA2 (35%). A total of 94 seedlings displayed RFP fluorescence, indicating successful co‐transformation of both T‐DNAs (60%). Among these 94 seedlings, 53 also exhibited GFP fluorescence, suggesting that both T‐DNAs were integrated as a single unit (34%). The remaining 41 plants showed only RFP fluorescence, indicating that both T‐DNAs were integrated separately (26%). Additionally, eight surviving plants exhibited only GFP fluorescence (~5%), suggesting the integration of T‐DNA2 along with the intervening region but without complete integration of T‐DNA1, which carries the GOI, DsRED (Figure 4).

When Arabidopsis seeds were sown on selection media after floral‐dip transformation, a large number of them germinated on the selection media but did not survive beyond the early seedling stage. While the exact percentage could not be determined, fluorescence analysis of these non‐surviving seedlings revealed various integration patterns. Some seedlings exhibited only RFP fluorescence, indicating integration of T‐DNA1 alone, while others displayed both red and green fluorescence, suggesting integration of T‐DNA1 and the intervening region but not complete integration of T‐DNA2. Additionally, some seedlings showed only GFP fluorescence, indicating integration of the intervening region without full integration of either T‐DNA1 or T‐DNA2. These observations highlight multiple patterns of T‐DNA integration in Arabidopsis, including T‐DNA1 alone (GOI+/GFP−/SMG−), T‐DNA2 alone (GOI−/GFP−/SMG+), T‐DNA1 and T‐DNA2 integrated separately (GOI+/GFP−/SMG+), T‐DNA1 and T‐DNA2 integrated as a single unit (GOI+/GFP+/SMG+), T‐DNA1 and the intervening region without complete integration of T‐DNA2 (GOI+/GFP+/SMG−), T‐DNA2 and the intervening region without complete integration of T‐DNA1 (GOI‐/GFP+/SMG+) and integration of the intervening region alone without complete integration of T‐DNA1 or T‐DNA2 (GOI−/GFP+/SMG−) (Figure 4).

In contrast, only two integration patterns—T‐DNA2 alone (GOI−/GFP−/SMG+) and T‐DNA1 and T‐DNA2 integrated separately (GOI+/GFP−/SMG+)—were observed in tobacco, tomato and lettuce. This is likely due to the different transformation methods used. In these species, transformation involved the regeneration of new shoots from leaf or cotyledon explants on selection media, which ensured that all surviving plants carried T‐DNA2 with SMG. Consequently, integration patterns lacking SMG were not detected. Furthermore, unlike in Arabidopsis, the integration patterns ‘GOI−/GFP+/SMG+’ and ‘GOI+/GFP+/SMG+’, which include SMG, were also absent in these species. However, small patches of green fluorescence were observed in some callus tissue across all three species, suggesting the occurrence of different integration events in callus (Figure S1).

### Agrobacterium strain significantly influences co‐transformation efficiency, but not vector backbone integration

The high incidence of co‐transformation of the two T‐DNAs as a single unit (34%, GOI+/GFP+/SMG+ plants) in Arabidopsis after transformation with pRED‐AN‐MF3 was unexpected, as no such events were observed in tobacco, tomato, or lettuce. Since Arabidopsis was transformed using Agrobacterium strain GV3101, while the other species were transformed with strain LBA4404, we investigated whether the observed differences in integration patterns were due to the Agrobacterium strain used. Initially, we attempted Arabidopsis transformation with strain LBA4404, but even after multiple transformation attempts, we were unable to recover enough transgenic seedlings for analysis. To further assess whether the Agrobacterium strain influenced integration patterns, we transformed tobacco with pRED‐AN‐MF3 using strain GV3101.

Following transformation, 300 regenerating shoots were randomly selected and analyzed for RFP fluorescence. Only 27 of 300 shoots (9%) displayed red fluorescence, which was significantly lower than the 19% co‐transformation efficiency observed when using strain LBA4404. A Pearson's chi‐squared test confirmed that this difference in co‐transformation frequency between GV3101 and LBA4404 strains was statistically significant (*χ* ^2^(1) = 9.74, *P* = 0.0018). PCR genotyping of 24 co‐transformed plants confirmed the presence of both T‐DNA1 and T‐DNA2. PCR amplification of the intervening region revealed that none of the plants exhibited linked integration (0%), consistent with the results obtained using LBA4404. Furthermore, no GFP expression was detected in any of the 300 analyzed transgenic shoots.

### Marker‐free plants were recovered in the T1 generation

We analyzed marker segregation in the T1 generation of tobacco plants transformed with vectors pRED‐AN‐MF1, pRED‐AN‐MF2, and pRED‐AN‐MF3. For each vector, two T0 lines that were positive for GOI and SMG, but negative for the intervening region were selected and grown in soil. T0 plants were grown under controlled growth room conditions, where the absence of external pollinating agents ensured self‐pollination for T1 seed production. Seeds were collected, and 40 T1 seedlings from each line were raised to assess the percentage of marker‐free plants. PCR analysis was conducted to detect the presence of GOI and SMG in the T1 generation (Table 2).

**Table 2:** Marker segregation in the T1 generation of tobacco plants transformed with double T‐DNA vectors: *χ* ^2^ values and associated *P*‐values are shown in the final columns

T0 line	GOI+/SMG+	GOI−/SMG+	GOI+/SMG−	GOI−/SMG−	Chi‐squared (df = 3)	*P*‐value
pRED‐AN‐MF1 L#8	31	7	2	0	9.78	0.021
pRED‐AN‐MF1 L#10	17	13	6	4	6.58	0.087
pRED‐AN‐MF2 L#4	34	5	0	1	15.11	0.002
pRED‐AN‐MF2 L#9	29	1	8	2	7.64	0.054
pRED‐AN‐MF3 L#4	33	3	1	3	13.33	0.004
pRED‐AN‐MF3 L#1	35	2	1	3	16.28	0.001

*P*‐values <0.05 indicate statistically significant deviation from expected Mendelian segregation.

Segregation analysis revealed four types of transgenic plants: GOI+/SMG+, GOI−/SMG+, GOI+/SMG−, and GOI−/SMG− (Table 2). In all analyzed lines, the majority of plants were GOI+/SMG+ (43–88%), while the proportions of GOI−/SMG+ (3–33%), GOI+/SMG− (0–20%), and GOI−/SMG− (0–10%) were significantly lower. Chi‐squared goodness‐of‐fit tests were conducted to assess whether the T1 progeny of each T0 line followed the expected 9:3:3:1 segregation ratio, assuming independent assortment of two unlinked loci. Four of the six T0 lines—pRED‐AN‐MF1 L#8, pRED‐AN‐MF2 L#4, pRED‐AN‐MF3 L#1, and L#4—showed statistically significant deviations from the expected ratio (p < 0.05). The remaining two lines (MF1 L#10 and MF2 L#9) did not significantly deviate.

**Figure 5:**
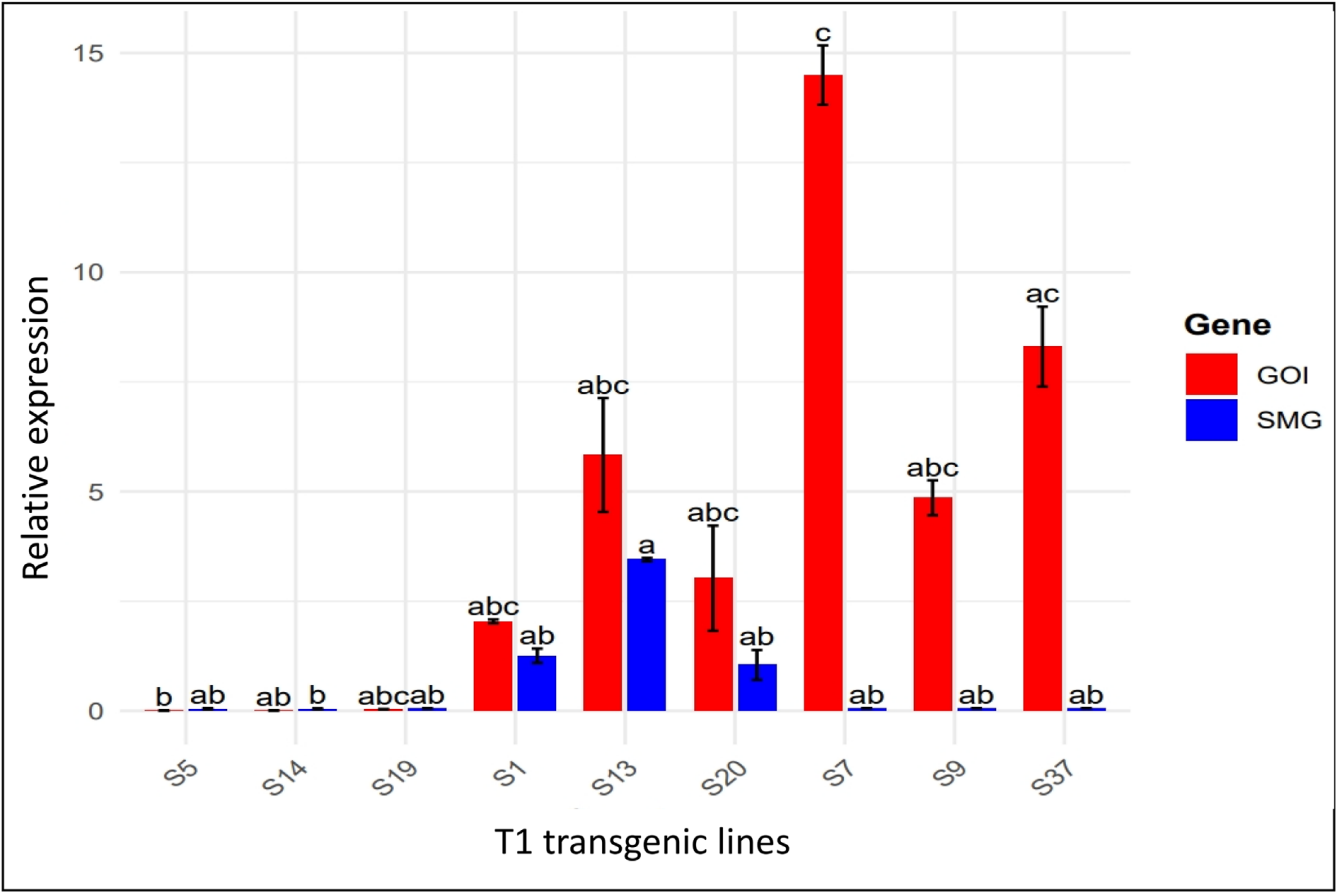
Relative expression of SMG and GOI in T 1 transgenic plants of pRED‐AN‐MF 1 L#10. Quantitative PCR (qPCR) analysis showing the relative expression of the transgenes: NPT II (SMG, blue) and DsRED (GOI, red) across different T1 transgenic lines. Transgenic tobacco lines S5, S14 and S19 were PCR negative for both the genes (GOI−/SMG−), lines S1, S13 and S20 were PCR positive for both the genes (GOI+/SMG+) and lines S7, S9 and S37 were PCR positive for GOI and negative for SMG (GOI+/SMG−). qPCR analysis was performed by 2^−ΔΔCT^ method using actin as the reference gene. Bars represent mean expression ± standard deviation from three biological replicates. Different letters indicate statistically significant differences among samples for each gene (Dunn's test, Bonferroni‐adjusted *P* < 0.05).

To confirm the segregation of the SMG from the GOI in the T1 generation, quantitative real‐time PCR (qRT‐PCR) was performed on selected T1 plants derived from a single T0 line. The qRT‐PCR results showed no detectable SMG expression in plants that were GOI‐positive and SMG‐negative (GOI+/SMG−). As expected, no expression of either gene was observed in GOI−/SMG− plants, while both genes were expressed in GOI+/SMG+ plants. These results indicate that the marker‐segregated lines identified through PCR and qPCR do not carry any amplifiable or transcriptionally active copies of the SMG (Figure 5).

## DISCUSSION

### The likelihood of linked integration is influenced by the distance between the T‐DNAs in the vector

Several natural Agrobacterium strains harbor multiple T‐DNA regions on their native Ti or Ri plasmids, which can be transferred independently into plant cells and often integrate as closely linked tandem or inverted repeats (Gelvin, 2003; Otten, 2021). Previous studies have reported high linked integration rates for double T‐DNA vectors with short intervening regions (Leng et al., 2020). Our analysis confirmed that shorter distances between T‐DNAs increase this likelihood. Co‐transformation efficiency, based on selection media growth, RFP expression, and PCR analysis, was highest in pRED‐AN‐MF1, followed by pRED‐AN‐MF2 and pRED‐AN‐MF3. However, PCR and sequencing of the intervening region revealed that many co‐transformed plants from pRED‐AN‐MF1 and pRED‐AN‐MF2 carried both T‐DNAs as a single unit, making them unsuitable as marker‐free candidates. These findings highlight the importance of genotyping the intervening region to differentiate between separate and linked T‐DNA integration and suggest that previously reported high co‐transformation efficiencies may partly result from unintended linked integration.

While several studies have examined the patterns of T‐DNA integration after co‐transformation (De Neve et al., 1997; Komari et al., 1996; Lee et al., 2004; Miller et al., 2002; Palanichelvam et al., 2000; Stahl et al., 2002) and the impact of T‐DNA size on co‐transformation efficiency (McCormac et al., 2001; Park et al., 2000; Xi et al., 2018), we were unable to find any research that specifically investigates the effect of the intervening region length on linked integration of T‐DNAs. Our findings suggest that maintaining an ~3 kb intervening region optimizes independent integration. Incorporating a reporter gene in this region effectively identified linked integration. Unlike Chen et al. (2005), which used a GFP cassette within the SMG T‐DNA to identify marker‐segregating T1 lines, our approach enables early detection of marker‐free candidates in T0, significantly reducing the number of plants requiring further analysis.

Some transgenic lines exhibited physically unlinked but closely integrated T‐DNAs, resulting in unexpected amplicon sizes. Notably, our primers were designed to detect only tandem‐oriented integrations, meaning inverse or other close integration events may have been missed due to PCR and primer limitations. Therefore, it is crucial to consider these possibilities when selecting potential marker‐free plants in the T0 generation.

The high incidence of linked co‐integration of the two T‐DNAs in our study suggests the potential for large VB integrations, as T‐DNA 2 could be considered part of the VB for T‐DNA 1 and vice versa. This co‐integration implies that significant VB segments may be incorporated during Agrobacterium‐mediated transformations, a phenomenon previously reported in Arabidopsis and tobacco transgenic plants due to inefficient termination at the left border (LB), leading to read‐through (Caplan et al., 1985; De Buck et al., 2000; Kononov et al., 1997; Lee et al., 2004). While many studies on transgenic plants focus on genetic elements within the T‐DNA, it is equally important to consider the impact of sequences adjacent to the T‐DNA borders on plant growth and development. This concern is further amplified by the presence of bacterial selection markers and origins of replication near T‐DNA borders in many plant transformation vectors, which may pose regulatory challenges for the release of transgenic plants.

### Orientation of two T‐DNAs influences co‐transformation efficiency and their integration patterns

Previous studies have shown that the relative orientation of two T‐DNAs can influence the frequency of linked integration, with a switch from tandem (RB‐LB‐RB‐LB) to inverse (LB‐RB‐RB‐LB) orientation significantly reducing linked co‐integration and increasing the likelihood of obtaining marker‐free transgenic plants in the T1 generation (Leng et al., 2020).

However, our results revealed a different trend. Transgenic tobacco shoots with the inverse T‐DNA orientation (pRED‐AN‐MF2‐i) had a lower percentage of successful co‐transformation compared to those with the tandem orientation (pRED‐AN‐MF2). Additionally, PCR analysis of the intervening region showed a significantly higher frequency of linked co‐integration in pRED‐AN‐MF2‐i T0 plants. Some plants exhibited a smaller amplicon, inconsistent with the expected 1673 nt size, suggesting close integration of individual T‐DNAs in an inverse orientation. Similar observations were made by Leng et al. (2020), who also detected smaller fragments inconsistent with the expected intervening region size when using a double T‐DNA vector with inversely oriented T‐DNAs.

T‐DNA transfer is generally believed to be a directional process that begins at RB and terminates at LB (Zambryski, 1988). However, several studies have demonstrated that DNA transfer can also initiate at the LB (De Buck et al., 2000; Kuraya et al., 2004; Ramanathan & Veluthambi, 1995; van der Graaff et al., 1996). Additionally, research suggests that the RB can function as a termination site for DNA transfer, with low efficiency (Kim et al., 2003). Studies have also reported that the RB can frequently initiate integration of the entire vector despite the presence of an LB (Miranda et al., 1992). Moreover, previous research has shown that initiation from both borders in the reverse orientation is possible, though at a greatly reduced frequency (Horsch & Klee, 1986; Wang et al., 1984). Close integration of multiple T‐DNA in either orientation with respect to each other has also been reported in multiple studies (De Block & Debrouwer, 1991; De Neve et al., 1997; Radchuk et al., 2005).

The presence of the intervening region suggests multiple possible integration patterns, including reverse initiation at LB1 with read‐through RB1 and RB2, terminating at LB2, or reverse initiation at LB2 with read‐through RB2 and RB1, terminating at LB1. Another possibility is the independent integration of the two T‐DNAs in close proximity in an inverse orientation, or initiation at RB1 or RB2 leading to the integration of the entire vector. The primers used for PCR were positioned at least 150 nt away from the T‐DNA borders, making it impossible to detect linked co‐integration through initiation at RB1 in the reverse orientation with read‐through RB2 and termination at LB2, as well as initiation at RB2 in the reverse orientation with read‐through RB1 and termination at LB1. However, the possibility of such integrations still existing in the transgenic plants cannot be excluded. Additionally, independent insertions in a tandem orientation may also be present, which went undetected due to the specific primers designed to detect integrations in inverse orientations.

These results emphasize the necessity of PCR analyses with strategically placed primers to uncover additional integration patterns, as any closely integrated events would not be ideal for recovering marker‐free plants. Our findings indicate that using T‐DNAs in an inverse orientation results in distinct patterns of close integration, differing from previous reports (Leng et al., 2020).

### Species‐specific differences in co‐transformation efficiency and T‐DNA integration

When we evaluated the co‐transformation efficiency of two T‐DNAs in lettuce and tomato using vector pRED‐AN‐MF3 and Agrobacterium strain LBA4404, we observed variable co‐transformation efficiency. Importantly, we did not observe GFP fluorescence in any of the shoots regenerated in selection media indicating the absence of the integration of the intervening region. This confirmed that vector pRED‐AN‐MF3 is ideal for developing marker‐free transgenic plants in this species.

When the same vector was introduced into Arabidopsis via floral dip transformation using Agrobacterium strain GV3101, several distinct patterns of T‐DNA and VB integration were observed. A significant percentage of surviving plants exhibited RFP and GFP fluorescence, indicating linked integration of the two T‐DNAs. Seven different integration patterns were identified, suggesting multiple possible integration mechanisms, including initiation at RB1 and termination at LB1, initiation at RB2 and termination at LB2, initiation at RB1 with read‐through of LB1 and termination at LB2, initiation at LB1 with read‐through of RB2 and termination at LB2, initiation at RB1 with read‐through of LB1 and potential termination at RB2, and initiation at LB1 with termination at RB2. Additionally, the possibility of reverse initiation from the borders may have further contributed to these observed patterns.

Compared to Arabidopsis, lettuce, tomato, and tobacco showed fewer integration patterns, likely due to differences in transformation methods. In these species, only plants carrying the SMG survived in the selection media, limiting the variety of observed integration patterns. Of the four SMG‐carrying integration patterns observed in Arabidopsis, only two (GOI−/GFP−/SMG+, GOI+/GFP−/SMG+) were observed in these species. The other two patterns (GOI+/GFP+/SMG+ and GOI−/GFP+/SMG+) were not observed in any of the surviving shoots, even though GFP was observed in some callus tissue in all the species.

Transformation experiments in tobacco using vectors pRED‐AN‐MF1 and pRED‐AN‐MF2 demonstrated that LB read‐through can occur, resulting in linked co‐integration of the two T‐DNAs, especially when the intervening region is relatively short. Combined with the results from pRED‐AN‐MF3 transformations in tobacco, lettuce, and tomato, this suggests that the specific factors influencing transformation—such as explant type, Agrobacterium strain, and the regeneration process—do not favor the integration of large VB sequences or initiation from the LB. As a result, the two T‐DNAs are more likely to integrate separately in these species.

### Transformation target cells influence vector backbone integration

We observed a higher incidence of linked integration and VB integration after transforming Arabidopsis with pRED‐AN‐MF3 compared to transformations using the same plasmid in tobacco, lettuce, and tomato. To evaluate whether the Agrobacterium strain influenced the outcomes, we attempted Arabidopsis transformation using strain LBA4404. However, no transgenic seedlings were recovered, consistent with previous reports indicating that LBA4404 has significantly lower transformation efficiency in Arabidopsis compared to strain GV3101 (Oltmanns et al., 2010). Hence, tobacco was transformed with pRED‐AN‐MF3 using strain GV3101 for comparison.

The results showed that GV3101 significantly reduced co‐transformation efficiency in tobacco compared to LBA4404. However, as with LBA4404‐transformed tobacco, no GFP expression was observed in any transgenic shoots. PCR analysis of confirmed co‐transformants detected both T‐DNAs, but no linked co‐integration, suggesting that while the Agrobacterium strain affects co‐transformation efficiency, it does not significantly influence large VB integrations or linked integration.

Similar findings were reported in a study comparing the effects of Agrobacterium strain and plasmid origin of replication on transformation efficiency and VB integration in Arabidopsis and maize (Oltmanns et al., 2010). VB integration rates varied significantly between the two species, despite using the same strain (GV3101) and plasmid origin of replication. In Arabidopsis, transformed via the floral‐dip (FD) method, approximately 65% of transgenic plants exhibited VB integration, whereas in maize embryo transformation, the VB integration rate was around 45% (Oltmanns et al., 2010). These findings, along with our results, indicate that VB integration is influenced by factors beyond Agrobacterium strain and plasmid origin of replication. Further studies in Arabidopsis have also demonstrated that the integration of multiple T‐DNA copies after FD transformation, compared to root transformation, is not due to the Agrobacterium strain, T‐DNA vector copy number in the transforming bacteria, or plant genotype, but rather the effect of transformation target cells (De Buck et al., 2004; De Buck et al., 2009; Marjanac et al., 2008).

A key distinction between transformations in tobacco, tomato, lettuce, and Arabidopsis is the type of cells targeted for transformation. In tobacco, tomato, and lettuce, transformation occurs in somatic cells, with transgenic plants regenerated through de novo shoot organogenesis. In contrast, Arabidopsis FD transformation primarily targets germline cells. It has been reported that the host factors essential for several stages of T‐DNA integration in somatic cells, including the duplication of the T‐strand into double‐stranded DNA and the illegitimate recombination of the T‐DNA molecule into double‐stranded breaks, differ from those required in germline cells (Citovsky et al., 2007; Gelvin, 2000; Gelvin, 2008; Mysore et al., 2000). Considering that certain gene products involved in DNA metabolism are believed to be more abundant in germline cells than in somatic tissues (Mysore et al., 2000), it may be possible that specific T‐DNA replication processes exist in female gametocytes, leading to changes in copy number and integration patterns.

Additionally, T‐DNA integration into the plant genome is influenced by the epigenetic landscape of the host cell (Gelvin, 2021; Shilo et al., 2017). Germline and somatic cells exhibit markedly different chromatin architectures—germline cells undergo dynamic reprogramming to reset epigenetic marks during gametogenesis, whereas somatic cells maintain more stable chromatin to preserve their differentiated state (Rose et al., 2013). These variations in chromatin accessibility between cell types can significantly impact T‐DNA integration patterns. High‐throughput studies have further demonstrated that the chromatin context plays a critical role in both the efficiency of integration and the selection of integration sites (Magori & Citovsky, 2011; Shilo et al., 2017).

Our results suggest that the transformation of somatic cells limits large VB integrations, whereas the transformation of germline cells enhances them. Notably, we observed GFP fluorescence in some growing callus tissue across all three species, indicating VB integration. This further supports the idea that plant factors involved in DNA replication and cell division may play a role in VB integration.

It was unexpected that GV3101, the more virulent strain, exhibited lower co‐transformation efficiency than the less virulent LBA4404. Studies indicate that GV3101 and LBA4404 differ in their ability to elicit plant defense responses upon infection (Li et al., 2017; Sheikh et al., 2014), and host compatibility and response to different Agrobacterium strains may play a key role in the co‐transformation of multiple T‐DNAs. GV3101's heightened virulence could activate stronger plant defense mechanisms following the successful delivery and integration of one T‐DNA, potentially reducing the likelihood of a second integration event. In contrast, LBA4404 may trigger weaker defense responses, thereby permitting a higher frequency of co‐transformation. This hypothesis, however, remains to be experimentally validated in future studies.

### The number of marker‐free plants recovered in the T1 generation was lower than anticipated

To determine whether PCR genotyping of the intervening region between two T‐DNAs is a reliable indicator for identifying potential marker‐segregating plants in the T0 generation, we analyzed T1 plants derived from T0 lines that were positive for the GOI and SMG but negative for the intervening region. These T0 lines were transformed with vectors pRED‐AN‐MF1, pRED‐AN‐MF2, and pRED‐AN‐MF3. Marker‐free plants were identified in all but one analyzed line, confirming that PCR genotyping is a reliable indicator for selecting these plants. qRT‐PCR analysis of the identified marker‐free lines showed high expression levels of the GOI, while no detectable expression of the SMG was observed, confirming the absence of transcriptionally active copies of the SMG.

Despite selecting T0 plants that were PCR‐negative for the intervening region, a significant percentage of T1 plants still carried both GOI and SMG. Previous studies in Arabidopsis have shown that multiple T‐DNAs can integrate into the same genomic locus, often in direct, head‐to‐head, or tail‐to‐tail orientations, leading to their linked inheritance (Radchuk et al., 2005). DNA integration sites have been suggested to act as “hot spots” for additional T‐DNA insertions, a notion supported by studies in barley where multiple T‐DNAs were found to integrate preferentially within specific regions of the BARE‐1 retrotransposon (Radchuk et al., 2005; Stahl et al., 2002). In Arabidopsis, a sequence‐level bias favoring T‐DNA integration into AT‐rich regions has also been reported (Brunaud et al., 2002). Notably, a similar tendency for integration at the same locus has been observed even when two T‐DNAs are delivered on separate plasmids via different Agrobacterium strains, indicating that physical linkage can occur independently of the delivery method (De Block & Debrouwer, 1991).

Such linked co‐integrations at nearby genomic locations—potentially at distances or orientations undetectable by the PCR primers used—could explain the unexpectedly high number of T1 plants retaining both GOI and SMG. To improve the efficiency of selecting potential marker‐free plants in the T0 generation, future approaches could include using more sensitive long‐range PCRs aiming to detect multiple T‐DNA insertions in different orientations.

## CONCLUSIONS

This study underscores the critical impact of spacer length and T‐DNA orientation on co‐transformation efficiency and integration patterns when using double T‐DNA vectors for generating marker‐free transgenic plants. Our findings demonstrate that shorter spacer regions between T‐DNAs significantly increase the risk of linked integration, while an intervening sequence of approximately 3 kb effectively reduces this risk. Contrary to prior reports, inverse T‐DNA orientation did not minimize linked integration but instead increased closely spaced and physically linked insertions. Additionally, transformation outcomes varied across plant species, and VB integrations were found to be influenced by factors beyond the Agrobacterium strain. Our results point towards the potential influence of transformation target cells in VB integrations. The inclusion of a reporter gene within the intervening region proved to be a valuable tool for the early identification of linked T‐DNA integration events in T0 plants, thereby streamlining the recovery of marker‐free lines. Collectively, our results underscore the importance of vector design and transformation strategy for the efficient and precise generation of marker‐free transgenic plants.

## MATERIALS AND METHODS

### Construction of double T‐DNA vectors

To develop double T‐DNA constructs, we selected vector pRI 201‐AN (Takara Bio, San Jose, CA, USA), carrying SMG (NPT II) for kanamycin resistance. DsRED2 gene from plasmid pEGB 35S:DsRed:Tnos (Addgene no. 68220) was selected as the GOI. The GOI was amplified using primers RED_FRAG_F/R and cloned into the vector linearized with NdeI and SalI (NEB) via In‐Fusion cloning (Takara Biosciences, San Jose, CA, USA, www.takarabio.com). Successful cloning was confirmed by PCR and sequencing, resulting in the recombinant vector pRED‐AN. All cloning procedures were performed using the In‐Fusion HD Cloning Plus kit (Takara Bio), and all primers used are listed in Table S1.

Using pRED‐AN as the base vector, we constructed four double T‐DNA plasmids, each differing in either the distance between the two T‐DNAs or their relative orientation. In these constructs, T‐DNA1 carried the gene of interest (GOI, DsRED2), while T‐DNA2 contained the selectable marker gene (SMG, NPT II).

#### pRED‐AN‐MF1

A 345 nt DNA segment was commercially synthesized, containing both left (LB) and right (RB) border sequences. The LB and RB were separated by 119 nt (Figure S2) and inserted after GOI and before SMG, using primers BORDER_FRAG_F/R to amplify the fragment and primers pRED‐AN_VEC_F/R to linearize the vector. The resulting plasmid pRED‐AN‐MF1 has two T‐DNAs in tandem orientation (RB1‐LB1‐RB2‐LB2) separated by 119 nt (Figure 1).

#### pRED‐AN‐MF2

To increase the distance between the two T‐DNAs, a 1048 nt filler region was amplified from pCAMBIA1302 using primers FILLER_FRAG_F/R. This fragment was inserted into pRED‐AN‐MF1 after linearizing the plasmid using primers pRED‐AN_VEC_F2/R2, increasing the distance between the two T‐DNAs to 1167 nt. The resulting plasmid, pRED‐AN‐MF2, retained the tandem orientation (RB1‐LB1‐RB2‐LB2) (Figure 1).

#### pRED‐AN‐MF3

To further increase the intervening distance, a 1907 nt enhanced GFP (eGFP) cassette, consisting of the CaMV 35S promoter, GFP coding sequence, and nopaline synthase terminator, was amplified from pGFPGUSPlus (Addgene no. 64401) using primers GFP_FRAG_F/R. The fragment was inserted into pRED‐AN‐MF2, which had been linearized with primers pRED‐AN_VEC_F3/R3. The resulting plasmid, pRED‐AN‐MF3, contained two T‐DNAs in tandem orientation (RB‐LB‐RB‐LB) separated by a 3074 nt intervening region harboring the GFP cassette (Figure 1).

#### pRED‐AN‐MF2‐i

To explore the effect of T‐DNA orientation, a 2516 nt fragment containing T‐DNA1 (GOI) was amplified from pRED‐AN‐MF2 using primers GOI_FRAG_F/R. The vector was linearized with primers pRED‐AN_VEC_F4/R4, and the T‐DNA1 fragment was reinserted in an inverse orientation (LB1‐RB1‐RB2‐LB2). This modification resulted in the pRED‐AN‐MF2‐i plasmid, where T‐DNAs were separated by 1228 nt (Figure 1).

### Plant materials and growth conditions

Tobacco (*Nicotiana tabacum* L.) cultivar Petit Havana SR1, tomato (*Solanum lycopersicum*) cultivar Micro‐Tom, lettuce (*Lactuca sativa*) cultivar Parris Island cos, and *Arabidopsis thaliana* genotype Columbia‐0 were used in this study. Seeds of all four species were purchased commercially and surface sterilized by suspending in 70% ethanol for 1 min, followed by treatment with 10% bleach for 20 min. After thorough washing with sterile distilled water, the sterilized seeds were plated onto germination medium (half‐strength Murashige and Skoog salt (½ MS) medium supplemented with 1% sucrose and 0.7% plant agar) for germination. Germination was initiated by incubating the seeds in darkness at 5°C for 2 days, followed by transfer to a light environment at ~22°C with a photoperiod of 16‐h light/8‐h dark for further growth. All reagents were purchased from Sigma (St. Louis, MO, USA).

### Agrobacterium‐mediated plant transformations

The four double T‐DNA vectors were individually introduced into *Agrobacterium tumefaciens* strain LBA4404 using the freeze–thaw transformation method (Chen et al., 1994). Tobacco was transformed with all four plasmids, while only pRED‐AN‐MF3 was transformed to the other species. To assess the impact of bacterial strain on co‐transformation efficiency and VB integration, tobacco was also transformed with pRED‐AN‐MF3 using Agrobacterium strain GV3101. For all species, the kanamycin concentration that prevents the regeneration of non‐transgenic shoots was determined from previous experiments.

#### Tobacco transformation

Leaf explants from 2‐month‐old tobacco seedlings were transformed following the protocol described by Clemente (2006). Transgenic plants were selected on tobacco shoot regeneration medium (TB‐SR) containing 3% MS media, 2 mg/L kinetin, and 1 mg/L IAA, supplemented with 100 mg/L kanamycin.

#### Tomato transformation

Cotyledon explants from 12‐day‐old tomato seedlings were transformed following the protocol described by Van Eck et al. (2019). Transgenic plants were selected on tomato shoot regeneration medium (SL‐SR) containing 3% MS media, 2 mg/L zeatin and 0.1 mg/L IAA, supplemented with 50 mg/L kanamycin.

#### Lettuce transformation

Cotyledon explants from 8‐day‐old lettuce seedlings were transformed following the protocol described by Roche et al. (2025). Transgenic plants were selected on lettuce shoot regeneration medium (LS‐SR) containing 3% MS media, 0.1 mg/L NAA, and 0.1 mg/L BAP, supplemented with 50 mg/L kanamycin. Lettuce was additionally transformed with a modified pRED‐AN‐MF3 vector in which the SMG cassette was replaced with the SMG cassette from vector PKSE 401 (Addgene no. 62202), conferring tolerance to kanamycin.

#### Arabidopsis transformation

For Arabidopsis transformations, *Agrobacterium tumefaciens* strain GV3101 was used based on previous reports (Clough & Bent, 1998; Ghedira et al., 2013). Arabidopsis transformation was carried out as per Zhang et al. (2006), and the transgenic T1 seeds were selected on ½ MS medium supplemented with 50 mg/L kanamycin.

All cultures were maintained under controlled conditions with a 16/8‐h light/dark cycle, temperatures of 26°C (day) and 24°C (night), and 70% relative humidity. Explants were subcultured onto fresh selection media every 14 days to promote regeneration and growth.

### PCR and fluorescence‐based genotyping of transgenic plants

To ensure that only transgenic shoots regenerated, a kanamycin concentration of 100 mg/L was used in the selection medium, based on prior experience showing that this level effectively suppresses regeneration from non‐transformed tobacco explants. Therefore, any shoot that regenerated under selection was presumed to have integrated T‐DNA2, which carries the SMG. The presence of T‐DNA1 in regenerating shoots was initially assessed by analyzing red fluorescent protein (RFP) expression under a Leica Thunder Imager System (Leica Microsystems Ltd., Switzerland) using LAS X software.

To confirm T‐DNA integration, genomic DNA was extracted from T0 and T1 transgenic plant leaves. PCR screening using AGRO_CHV_F/R primers was performed to identify and exclude any samples contaminated with residual Agrobacterium. Additionally, PCR with pRI_REPLI_F/pRI_REPLI_R primers, targeting an 850‐bp region within the pRi replication origin located outside the T‐DNA, was used to detect and eliminate samples with potential plasmid carryover. T‐DNA1 was detected by amplifying an 890 nt DsRED2 fragment (GOI_SEQ_F/R primers), while T‐DNA2 was confirmed by amplifying an 807 nt SMG fragment (NPT_SEQ_F/R primers).

To determine whether the two T‐DNAs were integrated as a single unit, PCR was performed using a forward primer (T‐DNA1_F1) in T‐DNA1 and a reverse primer (T‐DNA2_R1) in T‐DNA2. If both T‐DNAs were integrated as a single fragment, the expected PCR product sizes were 599 nt for pRED‐AN‐MF1, 1647 nt for pRED‐AN‐MF2, and 3554 nt for pRED‐AN‐MF3. For pRED‐AN‐MF2‐i transformed shoots, another PCR assay was conducted using T‐DNA1_F2 (forward) in T‐DNA1 and T‐DNA2_R1 (reverse) in T‐DNA2, yielding an expected fragment size of 1673 nt if the T‐DNAs were integrated as a single unit. Amplicons with unexpected sizes were analyzed by gel elution followed by sequencing using primers T‐DNA1_F1, T‐DNA1_F2, or T‐DNA2_R1. In pRED‐AN‐MF3‐transformed shoots, linked integration of T‐DNAs was further confirmed by visual inspection of GFP fluorescence, indicating the integration of the intervening region.

### Analysis of potential marker‐free plants in the next generation

From tobacco plants transformed with pRED‐AN‐MF1, pRED‐AN‐MF2, and pRED‐AN‐MF3, two PCR‐positive lines per construct—confirmed to contain both T‐DNA1 and T‐DNA2 but not integrated as a single unit—were selected for further study. T0 plants were grown under controlled growth room conditions, where the absence of external pollinating agents ensured self‐pollination for T1 seed production. Seeds from these lines were germinated on ½ MS medium without antibiotics, and 40 T1 seedlings from each line were genotyped to assess the presence of T‐DNA1 and T‐DNA2 using the primers described above.

### RNA isolation and quantitative real‐time PCR (qRT‐PCR)

To confirm marker segregation in the T1 generation, the expression profiles of the GOI and SMG were analyzed in marker‐segregated plants (GOI+/SMG−) and compared to those in GOI+/SMG+, GOI−/SMG+, and wild‐type (GOI−/SMG−) plants. For each category, three individual plants were analyzed. Leaf tissue was flash frozen in liquid nitrogen and total RNA was isolated using a TRIzol reagent (ThermoFisher Scientific, Waltham, MA, USA, Cat no. 15596026) based in‐house protocol. RNA reverse transcription was performed using QuantiTect Reverse Transcription Kit (Qiagen, Hilden, Germany). Gene‐specific primers were designed by Primer 3.0 software (Table S1). The qRT‐PCR reaction mixture was prepared using PowerUp™ SYBR™ Green Master Mix (Applied Biosystems™ by Thermo Fisher Scientific, Lithuania) and qRT‐PCR profiling was performed using a fluorescence quantitative instrument (StepOnePlus™ Real‐Time PCR System; Applied Biosystems™). Three biological replicates and three technical replicates were used for all qRT‐PCRs. Tobacco actin gene (GenBank accession number: X69885.1) was used as an internal reference (Schmidt & Delaney, 2010). The relative gene expression level was analyzed according to the 2^−ΔΔCT^ method (Livak & Schmittgen, 2001).

### Statistical analysis

Statistical analyses for Table 1 were conducted in R (version 4.2.3) using the dplyr and DescTools packages. Pearson's chi‐squared test was used to compare co‐transformation and linked integration frequencies across vector constructs, and Fisher's exact test was applied when chi‐squared assumptions were not met. For comparisons yielding significant overall differences, post hoc pairwise proportion tests with Bonferroni correction were performed. For Table 2, a chi‐squared (*χ* ^2^) goodness‐of‐fit test was used to assess whether the segregation patterns of each T_0_ line conformed to the expected Mendelian 9:3:3:1 ratio, assuming independent assortment of two unlinked heterozygous loci. A significance threshold of *p* < 0.05 was applied throughout. The qRT‐PCR results were subjected to Kruskal–Wallis test, followed by Dunn's post hoc test with Bonferroni correction.

## AUTHOR CONTRIBUTIONS

Conceptualization and experimental design—SG; experiments and data curation—MAN, ME, MR, MA and SG; Resources and supervision—KMAA, writing—original draft—SG, writing—review and editing—all authors. All authors have read and agreed to the submitted version of the manuscript.

## CONFLICT OF INTEREST

The authors declare that they have no known competing financial interests or personal relationships that could have appeared to influence the work reported in this paper.

## Supporting information


**Table S1.** Primers used in this study.
**Figure S1.** GFP florescence observed in callus tissue from selected tobacco explants transformed with the vector pRED‐AN‐MF3 using *Agrobacterium tumefaciens* strain LBA4404.
**Figure S2.** 345 nt DNA segment containing left and right border sequences (LB and RB, highlighted) CAGTACATTAAAAACGTCCGCAATGTGTTATTAAGTTGTCTAAGCGTCAATTTGTTTACACCACAATATATCCTGCCAAGTACTTTGATCCCGAGGGGAACCCTGTGGTTGGCATGCACATACAAATGGACGAACGGATAAACCTTTTCACGCCCTTTTAAATATCCGTTATTCTAATAAACGCTCTTTTCTCTTAGGTTTACCCGCCAATATATCCTGTCAAACACTGATAGTTTAAACTGAAGGCGGGAAACGACAATCTGATCATGAGCGGAGAATTAAGGGAGTCACGTTATGACCCCCGCCGATGACGCGGGACAAGCCGTTTTACGTTTGGAACTGACA.
**Figure S3.** Amplicon sequencing of T0 transgenic lines exhibiting PCR amplicons of unexpected sizes. PCR bands of unexpected size were gel‐purified and sequenced using primers T‐DNA1_F1 or T‐DNA1_F2. Representative results are shown. (A) *pRED‐AN‐MF1, line 22*: two T‐DNAs integrated in tandem with very short spacing; LB1 and RB2 are truncated. (B) *pRED‐AN‐MF2, line 11*: two T‐DNAs integrated in tandem with no intervening genomic sequence; LB1 and RB2, along with adjacent sequences, are truncated. (C) *pRED‐AN‐MF2‐i, line 4*: two T‐DNAs integrated in tandem separated by ~265 nt of tobacco genomic DNA; RB regions are truncated in both T‐DNAs.

## Data Availability

The dataset for Figure S3 is available in Figshare at DOI: https://doi.org/10.6084/m9.figshare.29986573.v1. All other relevant data is included in this article and its Supplementary Material; further inquiries can be directed to the corresponding author.
