# Supplementary material for: T‐DNA orientation, distance between two T‐DNAs, and the transformation target cells significantly impact vector backbone integration and efficiency of generating marker‐free transgenic plants in a co‐transformation system
Source: Plant J. 2025 Oct 7;124(1):e70510. doi: 10.1111/tpj.70510 (PMC12503609; doi:10.1111/tpj.70510)
Supplement: Supplementary file 1 — Table S1. Primers used in this study. Figure S1. GFP florescence observed in callus tissue from selected tobacco explants transformed with the vector pRED‐AN‐MF3 using Agrobacterium tumefaciens strain LBA4404. Figure S2. 345 nt DNA segment containing left and right border sequences (LB and RB, highlighted) CAGTACATTAAAAACGTCCGCAATGTGTTATTAAGTTGTCTAAGCGTCAATTTGTTTACACCACAATATATCCTGCCAAGTACTTTGATCCCGAGGGGAACCCTGTGGTTGGCATGCACATACAAATGGACGAACGGATAAACCTTTTCACGCCCTTTTAAATATCCGTTATTCTAATAAACGCTCTTTTCTCTTAGGTTTACCCGCCAATATATCCTGTCAAACACTGATAGTTTAAACTGAAGGCGGGAAACGACAATCTGATCATGAGCGGAGAATTAAGGGAGTCACGTTATGACCCCCGCCGATGACGCGGGACAAGCCGTTTTACGTTTGGAACTGACA. Figure S3. Amplicon sequencing of T0 transgenic lines exhibiting PCR amplicons of unexpected sizes. PCR bands of unexpected size were gel‐purified and sequenced using primers T‐DNA1_F1 or T‐DNA1_F2. Representative results are shown. (A) pRED‐AN‐MF1, line 22: two T‐DNAs integrated in tandem with very short spacing; LB1 and RB2 are truncated. (B) pRED‐AN‐MF2, line 11: two T‐DNAs integrated in tandem with no intervening genomic sequence; LB1 and RB2, along with adjacent sequences, are truncated. (C) pRED‐AN‐MF2‐i, line 4: two T‐DNAs integrated in tandem separated by ~265 nt of tobacco genomic DNA; RB regions are truncated in both T‐DNAs. [file TPJ-124-0-s001.docx]

**Table S1. Primers used in this study.**

| Primer name | Sequence (5’-3’) |
| --- | --- |
| RED_FRAG_F | TCACTGTTGATACATATGGCCTCCTCCGAGAAC |
| RED_FRAG_R | TTCAGAATTGTCGACCTACAGGAACAGGTGGTGG |
| BORDER_FRAG_F | AACCTGTCGTGCCAGCAGTACATTAAAAACGTCCGCAATGTG |
| BORDER_FRAG_R | TCAACGTTGCGGTTCTGTCAGTTCCAAACGTAAAACGGCT |
| pRED-AN_VEC_F1 | GAACCGCAACGTTGAAGGAGC |
| pRED-AN_VEC_R1 | CTGGCACGACAGGTTTCCC |
| FILLER_FRAG_F | TGATCCCGAGGGGAAGTCTGGATCGCGAAAACTGT |
| FILLER_FRAG_R | ATGCCAACCACAGGGGGCTTGTTGCCCGCTTC |
| pRED-AN_VEC_F2 | CCCTGTGGTTGGCATGC |
| pRED-AN_VEC_R2 | TTCCCCTCGGGATCAAAGTAC |
| GFP_FRAG_F | GGTGAATCAGCTTGCATGCCTGCAG |
| GFP_FRAG_R | AGGTGCGCCCGATCTAGTAACATAGATGACACC |
| pRED-AN_VEC_F3 | AGATCGGGCGCACCTCTGGCAACCG |
| pRED-AN_VEC_R3 | GCAAGCTGATTCACCACTTGCAAAGTCCCG |
| GOI_FRAG_F | GATGAATTGTTTTAGTTCCCCTCGGGATCAAAGTAC |
| GOI_FRAG_R | TTTCGCGATCCAGACTCACATACAAATGGACGAACGGATAAACC |
| pRED-AN_VEC_F4 | GTCTGGATCGCGAAAACTGTGG |
| pRED-AN_VEC_R4 | CTAAAACAATTCATCCAGTAAAATATAATATTTTATTTTCTCCCAATCA |
| AGRO_CHV_F | CCGCTCTCCTGGCATCATCT |
| AGRO_CHV_R | GTCACGAATTGGCGCATCTG |
| pRI_REPLI_F | ATTTCCAGTTTGGCCTTGCT |
| pRI_REPLI_R | GCTCACAGCGAACATTGGTA |
| GOI_SEQ_F | CACGGGGGACTCTAGATACATCAC |
| GOI_SEQ_R | CTTAAGCACACAAGCTAGCTTTTTATTTGACAC |
| NPT_F | GATGGATTGCACGCAGGTTC |
| NPT_R | TCATTTCGAACCCCAGAGTCC |
| T-DNA1_F1 | GTTATCCGCTCACAATTCCACACA |
| T-DNA2_R1 | CTGATAGTGACCTTAGGCGACTTTTGAAC |
| T-DNA1_F2 | GGGGACCTGCAGGCA |
| GOI_RT_F | GCCACTACCTGGTGGAGTTC |
| GOI_RT_R | GGTGTAGTCCTCGTTGTGGG |
| NPT_RT_F | GATGGATTGCACGCAGGTTC |
| NPT_RT_R | CAGCCGATTGTCTGTTGTGC |
| NtACT9_RT_F | CCTGAGGTCCTTTTCCAACCA |
| NtACT9_RT_R | GGATTCCGGCAGCTTCCATT |


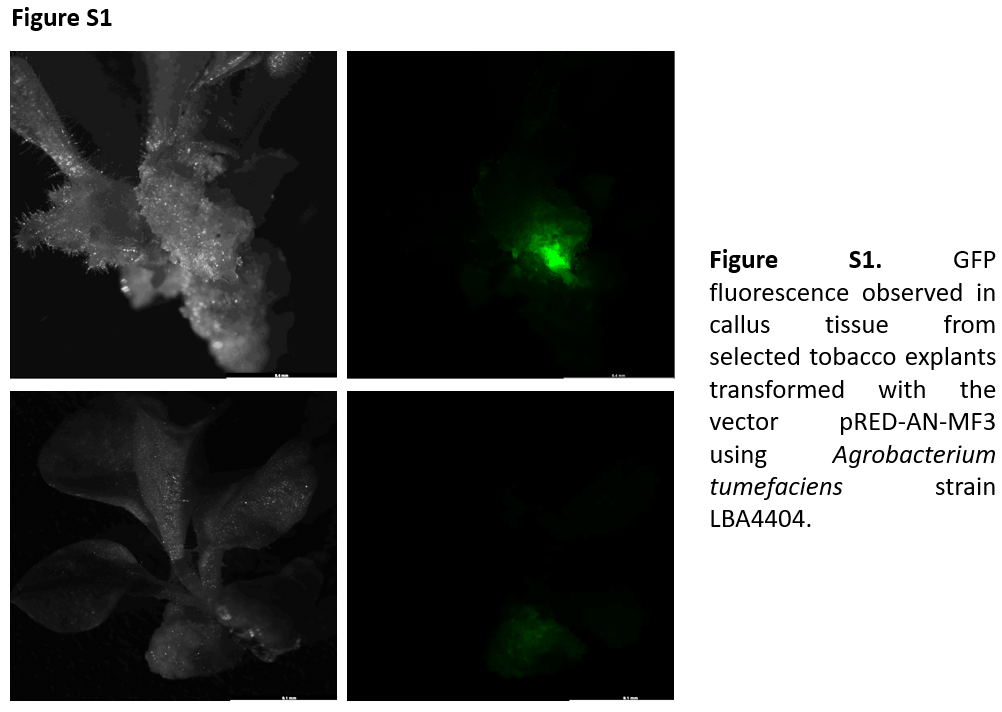


**Figure S2:** 345 nt DNA segment containing left and right border sequences (LB and RB, highlighted)

CAGTACATTAAAAACGTCCGCAATGTGTTATTAAGTTGTCTAAGCGTCAATTTGTTTACACCACAATATATCCTGCCAAGTACTTTGATCCCGAGGGGAACCCTGTGGTTGGCATGCACATACAAATGGACGAACGGATAAACCTTTTCACGCCCTTTTAAATATCCGTTATTCTAATAAACGCTCTTTTCTCTTAGGTTTACCCGCCAATATATCCTGTCAAACACTGATAGTTTAAACTGAAGGCGGGAAACGACAATCTGATCATGAGCGGAGAATTAAGGGAGTCACGTTATGACCCCCGCCGATGACGCGGGACAAGCCGTTTTACGTTTGGAACTGACA

**Figure S3: Amplicon sequencing of T0 transgenic lines exhibiting PCR amplicons of unexpected sizes.**
PCR bands of unexpected size were gel-purified and sequenced using primers T-DNA1_F1 or T-DNA1_F2. Representative results are shown. (A) *pRED-AN-MF1, line 22:* two T-DNAs integrated in tandem with very short spacing; LB1 and RB2 are truncated. (B) *pRED-AN-MF2, line 11:* two T-DNAs integrated in tandem with no intervening genomic sequence; LB1 and RB2, along with adjacent sequences, are truncated. (C) *pRED-AN-MF2-i, line 4:* two T-DNAs integrated in tandem separated by ~265 nt of tobacco genomic DNA; RB regions are truncated in both T-DNAs.


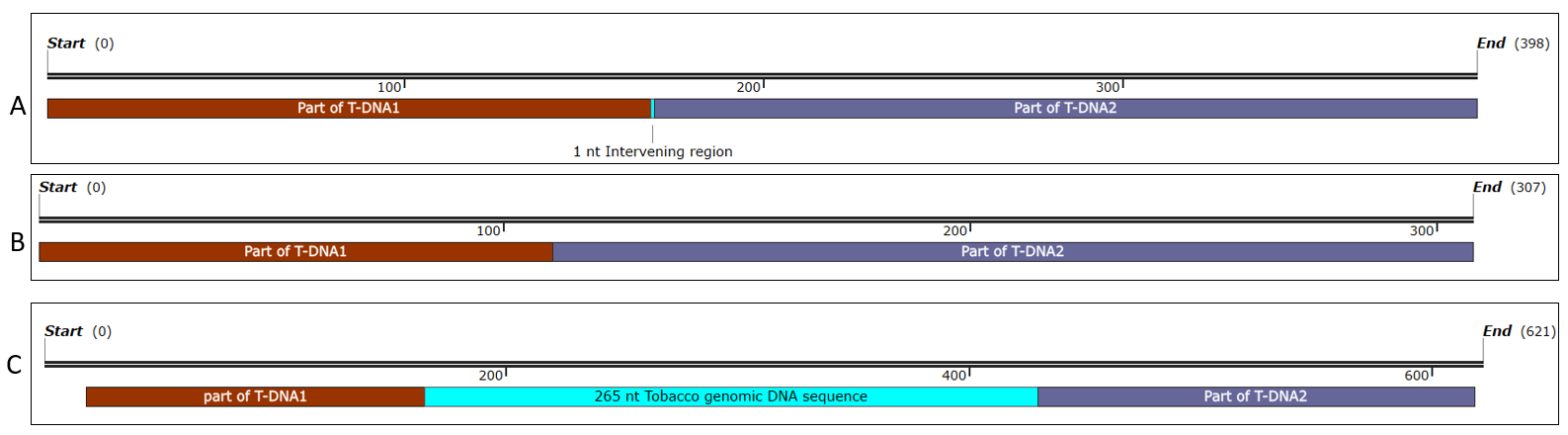


**Sequences for Figure S3 are available on Figshare at DOI: https://doi.org/10.6084/m9.figshare.29986573.v1.**
